# Electro-acupuncture with different current intensities to treat functional constipation: a study protocol for a randomized controlled trial

**DOI:** 10.1186/1745-6215-14-344

**Published:** 2013-10-22

**Authors:** Cui Hong Zheng, Guang Ying Huang, Xiao Hu Xu, Ying Wang, Ming Min Zhang, Wei Wang, Xiang Hong Jing, Bing Zhu

**Affiliations:** 1 Institute of Integrated Traditional Chinese and Western Medicine, Tongji Hospital, Tongji Medical College, Huazhong University of Science and Technology, Wuhan, Hubei 430030, China; 2 Department of Neurology, Tongji Hospital, Tongji Medical College, Huazhong University of Science and Technology, Wuhan, Hubei 430030, China; 3 Institute of Acupuncture and Moxibustion, China Academy of Chinese Medical Sciences, Beijing, 100700, China

**Keywords:** Acupuncture, Functional constipation, Study protocol

## Abstract

**Background:**

Functional constipation (FC) is highly prevalent in the general population of the world and has a substantial negative impact on the health-related quality of life of individuals. Many clinical trials have indicated that acupuncture is effective in the treatment of FC. However, the sample sizes of these previous studies were too small. Furthermore, there are no reports investigating the relationship between the stimulation parameter and the therapeutic effect. We therefore designed a multicenter randomized controlled trial to address these problems and hopefully provide a more conclusive answer to these questions.

**Methods:**

Participants will be included if they meet all of the following conditions: (1) diagnosed with functional constipation according to the Roman III standard; (2) aged between 18 and 65 years; (3) not taking any drugs that promote gastrointestinal movements at least during the 1 week prior to randomization; (3) willing to sign an informed consent form; (4) willing to return to the study site for their study visits. The participants will be randomly assigned to three groups in a 1:1:1 ratio: high current intensity group, low current intensity group, and mosapride citrate control group. The total study period is 9 weeks for each patient, 1 week for baseline, 4 weeks for treatment, and 4 weeks for follow-up. The primary outcome in this trial is the number of defecating events per week. The secondary outcomes will include the shape and properties of the stool, intensity of defecating difficulty, Patient Assessment of Constipation Quality of Life (PAC-QOL), MOS item Short Form health survey (SF-36), Self-Rating Anxiety Scale (SAS), and Self-Rating Depression Scale (SDS).

**Discussion:**

This study will provide significant evidence for the application of acupuncture in FC and will identify a suitable stimulation parameter for treatment.

**Trial registration:**

ClinicalTrials.gov ID: NCT01274793.

## Background

Functional gastrointestinal disorders (FGIDs) are very common diseases worldwide [1]. Functional constipation is a common type of FGIDs described by persistently difficult, infrequent, or incomplete defecation that does not meet the IBS criteria [2]. Surveys have shown that the prevalence of functional constipation in the general population of the US and Asia is 12%-19% [3] and 14% [4], respectively.

The mean annual cost per patient with constipation is estimated to be $7,522 [5]. There are significant economic costs associated with treating functional constipation, including direct costs associated with evaluation and treatment, as well as indirect costs such as missing school or work or diminished productivity. Therefore, chronic constipation has a substantial negative impact on the health-related quality of life of individuals [6].

To treat chronic constipation, a number of lifestyle modification activities are recommended, such as consumption of a high fiber diet, frequent exercise, and drinking plenty of fluids. Conventional treatments for functional constipation include stool softeners, bulking agents, osmotic and stimulant laxatives, and lubiprostone (a chloride channel activator). However, a systematic review of chronic constipation conducted by the American College of Gastroenterology (ACG) Task Force showed that there is little evidence to support the use of many of these agents, with the exceptions of osmotic laxative lactulose and polyethylene glycol, which were found to improve stool frequency and stool consistency [7]. However, the sideeffects of laxatives cannot be ignored. Many patients have reported the effectiveness of laxatives, but their effects disappear when usage of the drugs stops.

Consequently, many constipation patients ask for additional help. Acupuncture, an important part of traditional Chinese medicine (TCM) that dates back at least 3000 years, can cure disease because it can stimulate the body’s self-regulatory ability, which is characterized by integrity and ambidirectional dominance. Acupuncture has gained increased popularity in Western countries because of its convenience, safety, and unique therapeutic effects. Both ancient literature and clinical practice have showed that acupuncture has many advantages for treating functional diseases of the nervous system, motor system, and digestive system, especially for functional bowel diseases, such as functional constipation, diarrhea, and dyspepsia. A number of clinical trials have shown that acupuncture is effective in the treatment of functional constipation, although the sample sizes of these trials were small. Furthermore, the effectiveness of acupuncture for the treatment of childhood constipation has been widely admitted [8-10]. However, as far as adult constipation is concerned, there are no randomized controlled trials (RCTs) that conclusively demonstrate the effectiveness of acupuncture. Furthermore, there are no reports of the relationship between the stimulation parameter and the therapeutic effect. We therefore designed a multicenter RCT to address these problems and hopefully provide a more conclusive answer to these questions.

The primary objective of this study is to investigate whether electro-acupuncture can effectively treat functional constipation through a comparison with mosapride citrate. The secondary objective is to determine whether different stimulation parameters (current intensities) result in different therapeutic effects for managing this condition.

## Methods

### Study design

**Figure 1:**
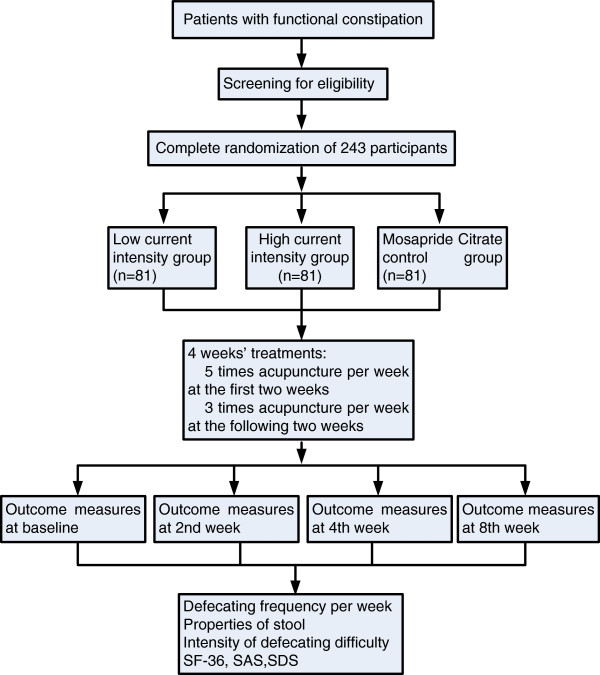
Trial flow chart.

A multicenter, randomized, parallel, controlled trial is currently being performed (Figure 1). Participants will be included from the following three hospitals: Affiliated Tongji Hospital of Huazhong University of Science and Technology, Affiliated Hospital of Hubei University of TCM, and Affiliated Hospital of Guangzhou University of TCM. Each research center must be in strict accordance with diagnostic standards, inclusion criteria and exclusion criteria.

The research protocol has been approved by the Clinical Trial Ethics Committee of Tongji Medical College, Huazhong University of Science and Technology (approval no. FWA00007304) and registered on the ClinicalTrials.gov protocol registration system (https://register.clinicaltrials.gov, ClinicalTrials.gov ID: NCT01274793). Approval to conduct this study has also been obtained from the involved hospitals and corresponding ethics committees. Before randomization, all participants will be fully informed and provided enough time to decide whether they will take part in this trial. The participants will be asked to sign a written informed consent based on their own will. Patients will be recruited to join the study only once and will not receive any monetary compensation for their participation.

### Inclusion criteria

Participants will be included if they meet all of the following conditions: (1) diagnosed with functional constipation according to the Roman III standard; (2) aged between 18 and 65 years; (3) not taking any drugs that promote gastrointestinal movements at least during the 1 week prior to randomization; (3) willing to sign an informed consent form; (4) willing to return to the study site for their study visits.

### Exclusion criteria

Participants will be excluded if they meet any of the following conditions: (1) unconsciousness, psychosis or failure to express subjective symptoms (acupuncture is an invasive intervention that requires 30 min per treatment; therefore, to ensure safety, we will not include these patients); (2) pregnant women or women in their lactation period (because it is still unclear whether acupuncture will lead to abortion or other side effects); (3) complicated with serious diseases of the heart, liver, kidney, and other organs; (4) bleeding disorders (acupuncture is invasive, and the procedure involves penetration of the skin; therefore, patients with bleeding tendency will be excluded); (5) previous participation in this study.

Patients have a choice whether to participate in the study, and they may withdraw their consent at any time. Their consent or lack thereof will not affect their deserved treatments.

### Recruitment strategies

Four strategies will be used to recruit participants with functional constipation: hospital-based recruiting, university-based recruiting, community-based recruiting, and television and newspaper advertisements. Hospital-based recruiting will involve the recruitment of participants in outpatient departments from the three hospitals, mainly from the digestion and acupuncture departments. Posters of this trial will be shown outside of the departments or hospitals to attract possible candidates. As to university or community recruiting, leaflets will be sent out to universities or communities near the research centers, and posters will be posted in the campus or community to enhance awareness. In addition, advertisements through TV, broadcasts, newspapers, and so on will be used to attract more patients.

### Sample size

Sample size calculation is based on a study by Camilleri:A placebo-controlled trial of prucalopride for severe chronic constipation [J]. N Engl J Med, 2008, 358 (22): 2344–2354 [11]. After drug treatment, the average defecation rate of patients with functional constipation was 2.6 times per week, with a standard deviation of 2.2. Furthermore, there was a 1.4-fold difference in the clinical effects between the drug and the placebo. Therefore, based on this study along with our preliminary test, the mean defecating frequency is 4.0 after acupuncture treatment, with a standard deviation of 3. To detect this difference, we need a minimum total sample size of 213 [71 for each group (α = 0.05, β = 0.10)] [12]. To allow for a loss of 15%, a total sample size of 243 (81 for each group) is required.

### Randomization and blinding

The participants will be randomly assigned to three groups through complete randomization at a 1:1:1 ratio equally among the three centers: high current intensity group, low current intensity group, and mosapride citrate control group. Therefore, each group in each center will have 27 participants. The randomization sequence will be generated using R2.0 software. A designated researcher will prepare the assignments in opaque envelopes in sequence.

The acupuncturists will only know of the grouping scheme just prior to the treatment to ensure that the trial will be as blinded as possible. The researchers will not be permitted to predict a patient’s assignment or change it after the patients are randomized. Blinded evaluation (the curative effect will be evaluated by a third party who does not know the grouping) and blinded statistical analysis will be emphasized during the data collection and analysis stage.

### Interventions

**Figure 2:**
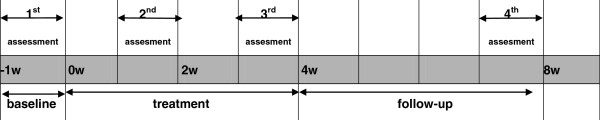
The total study period and the timepoint of evaluation.

The total study period will be 9 weeks for each patient, 1 week for baseline, 4 weeks for treatment, and 4 weeks for follow-up (5 to 8 weeks after randomization at 0 week) (Figure 2). Participants in the electro-acupuncture groups will receive 16 acupuncture treatments: five times per week (once a day for 5 days continuously, followed by a 2-day interval) during the first 2 weeks and three times per week (once every 2–3 days) during the following 2 weeks. Each session will last 30 min.

Acupoints Quchi (LI11) and Shangjuxu (S37) will be used on both sides, which is consistent with the principles of traditional acupuncture theories. Sterile, disposable needles made in Shanghai, China, will be used in this trial. The needles will be 25 to 50 mm in length and 0.3 mm in diameter. De qi sensation will be achieved in the acupuncture groups through lifting and thrusting movements combined with twirling and rotating the needles. After being acupunctured, the points will be punctured again using auxiliary needles 2 mm lateral to the first needle, to a depth of 2 mm, without manual stimulation. The auxiliary needles will be of the same brand, being 13 mm in length and 0.18 mm in diameter. The acupuncture needle and auxiliary needle of each point will be connected to form a circuit containing a HANS-200E stimulator (Nanjing Jisheng Co., China) for 30 min. The reason behind using the auxiliary needle is to guarantee the current confined in each acupoint. The stimulation parameters of electro-acupuncture in the high current intensity group will be an alternating wave with a frequency of 2/50 Hz; the current will be strong enough to reach the patients’ tolerance threshold value (1.0-1.8 mA, based on preliminary results). The frequency will be the same in the low current intensity group; while the current (0.2-0.7 mA) applied to this group will be relatively weak, it will be clearly perceived by the participants.

Participants in the control group will be orally administered 5 mg mosapride citrate tablets three times a day for 4 continuous weeks.

### Outcome measures

All of the participants in this trial will be asked to update constipation diaries from 1 week before randomization to 8 weeks after randomization. Only when they are eligible in the baseline phase for this study will the participants be asked to make diary entries from 0 to 8 weeks after randomization.

The primary outcome in this trial is the number of defecating events per week at baseline and at the 2nd, 4th and 8th week after randomization (Table 1 and Figure 2). Secondary outcomes include the following five aspects: shape and properties of stool, intensity of defecating difficulty, Patient Assessment of Constipation Quality of Life (PAC-QOL), MOS item Short Form health survey (SF-36), Self-Rating Anxiety Scale (SAS), and Self-rating Depression Scale (SDS). The first three of the secondary outcomes will be measured at baseline and at the 2nd, 4th and 8th week after randomization. The other secondary outcomes will be measured at baseline and at the 2nd and 4th week after randomization, although SF-36 will be measured only before randomization and 4 weeks after randomization. Additionally, the time of the first spontaneous defecation (from the end of the first intervention to the first spontaneous defecation) will be recorded.

**Table 1:** Trial process

	**Baseline**	**Treatment**			**Follow-up**
**Time**	−1w	0w	2w	4w	8w
**Assessment**		1st	2nd	3rd	4th
**Patients**					
Medical history	√				
Physical examination	√	√	√	√	
Laboratory examination	√			√	
Sign informed consent		√			
Randomization		√			
**Intervention and control**					
HCI group	16 sessions of acupuncture				
LOI group					
MC group	5 mg mosapride citrate tablet, three times a day				
**Outcomes**					
Number of defecating events per week		√	√	√	√
Shape and properties of stool		√	√	√	√
Intensity of defecating difficulty		√	√	√	√
PAC-QOL		√	√	√	√
SF-36		√		√	
SAS		√	√	√	
SDS		√	√	√	
The time of the first spontaneous defecation					
**Safety assessment**					
Safety of electro-acupuncture		√	√	√	
Adverse event		√	√	√	
**Trial evaluation**					
Patient’s compliance				√	
Rates of dropouts and withdrawals				√	

*HCI* high current intensity; *LCI* low current intensity; *PAC-QOL* Patient Assessment of Constipation Quality of Life; *SF*-36 MOS item Short Form health survey; *SAS* Self-Rating Anxiety Scale; *SDS* Self-rating Depression Scale.

### Patient safety

All patients participating in this trial will receive routine blood, urine, and stool tests as well as an electrocardiogram, blood biochemical tests (ALT, AST, BUN, and Scr), and colonoscopy prior to randomization. These tests will help identify and exclude patients who have serious heart, liver, and kidney diseases or other severe diseases. To exclude pregnant women, urine HCG or blood β-HCG will be tested for possible pregnancy. The patients will receive routine blood, urine, and stool tests after completing the treatment. These tests will help assess the risks associated with acupuncture or mosapride treatment.

### Quality control and trial monitoring

To guarantee the quality of the study, all acupuncturists and researchers will be required to undergo special training, including theoretical and practical lessons. They must master all of the details of this trial before performing it. For instance, they will be trained on how to use the randomization method, fill in the case report form, manipulate the needles and electro-acupuncture apparatus, etc. After attending all of the training classes and passing the training test, the researchers will be qualified to perform this trial. During the trial, all adverse events, including broken needles, bleeding, hematoma, fainting, serious pain, and local infection, are to be recorded during the treatment and the follow-up period. Serious adverse events will be immediately reported to the principal investigator. All details will be recorded, and rescue procedures will be initiated at once.

To ensure the quality of this trial, clinical monitor designated by the principle investigator will check all of the details of the process at regular intervals. Moreover, the monitor will check the authenticity of the data from each research centers.

### Trial evaluation

The trial evaluation will include patient compliance and the ratio of dropouts or withdrawals, which will be measured at the 4th week after randomization, and their reason will be recorded in time.

### Statistical analysis

Statistical analyses will be undertaken by the Department of Epidemiology and Biostatistics, School of Public Health, Tongji Medical College, Huazhong University of Science and Technology. Baseline characteristics of the subjects will be described by descriptive analysis and analyzed by analysis of variance, *χ*^2^-test, and others. With regard to the effects of intervention, the *χ*^2^-test will be applied to compare frequencies between groups, such as the number of defecating times per week; analysis of variance will be performed for comparison of continuous parameters between the groups (α = 0.05). All of the analyses will be based on both an intention-to-treat analysis (ITT) and a treated-per-protocol analysis (TPP).

## Discussion

Acupuncture has the characteristic of integral two-way adjustment and can regulate multiple body function disorders, such as constipation and diarrhea. The selected points can be the same or different. However, the quality of past studies has been poor, e.g., small sample size, no description of the methods for randomization, and no standardized acupuncture protocol, which may lead to performance bias. The project “Two-way adjusting effects of acupuncture for functional bowel disease and its mechanism” is sponsored and financed by the ‘973 Program’ administered by the Ministry of Science and Technology of China, which is the most important basic research program based on clinical practice. Twelve scientific institutes participate in this project. A multicenter, randomized, parallel controlled trial has been designed to confirm the effectiveness of acupuncture for functional constipation and functional diarrhea, and to explore the possible relationship of stimulus quantity and their effects. This article is just one part of this trial for addressing problems related to constipation.

The rationale behind the study of electro-acupuncture intensity is that different parameters of stimulation may cause different therapeutic effects for the same disease; the same parameters of stimulation may also bring different effects for different diseases. That is, some dysfunction may need mild stimulation, while others may need strong stimulation. In addition, different subjects, even different parts of one subject, can have different levels of sensitivity and acceptability. Therefore, the currents applied in the high- and low-intensity groups cannot be constant values, but instead, variable ranges. Based on the preliminary study, using an alternating wave of 2/50 Hz, the current reaching the patients’ tolerance threshold value ranges from 1.0-1.8 mA, while the current in the low-intensity group is relatively weak; a 0.2-0.7 mA current can be clearly perceived by the participants.

As for the rationale of acupoint selection, we screened acupoints through a systematic review of ancient books, acupuncture textbooks, and published articles. Meetings were held to further screen for a standardized acupuncture protocol and reach consensus among acupuncture experts in China. Based on the theory of acupuncture, Back-Shu and Front-Mu points (short for Shu-Mu points), which are located in the lower back or abdomen of the body, are the classic combination for the treatment of internal organ disease. Additionally, Hepoint (Quchi, LI11) and Lower Hepoint (Shangjuxu, S37) of the large intestine meridian are also positive points, which are respectively situated in the upper and lower limbs of the body. Nei Jing states: ‘He points are the best points to treat internal organ’s disease.’ However, our preliminary results have indicated that the effect of Shu and Mupoints in rats mainly excite gastrointestinal motility, while the effect of acupuncture in either Quchi or Shangjuxu is characterized by inhibiting motility. Therefore, the purpose of this study is to further validate the effect of acupuncture in Hepoints (Quchi and Shangjuxu) on functional constipation, as well as the relationship between the stimulation intensity and the resulting effect.

In this trial we are using mosapride as a positive control group rather than sham acupuncture as a negative control group. This setting is mainly based on the fact that any surface stimulation at acupoints, such as acupressure, can cause a physiological reaction or effect [13,14]. Thus, it is difficult to set a real non-active control for clinical acupuncture trials. As for mosapride, it is a gastroprokinetic agent that acts as a selective 5HT4 agonist. Mosapride accelerates gastric emptying and is used for the treatment of acid reflux, irritable bowel syndrome and functional dyspepsia [15]. Thus, mosapride also has the potential to treat functional constipation [16]. We therefore chose mosapride citrate as a positive drug control.

This study will provide significant evidence for the application of acupuncture in functional constipation and will be the first to identify a suitable parameter of stimulation for treatment.

## Trial status

This trial is currently recruiting participants. The first participant was included on 14 December 2011. To date (15 March 2013), 123 participants have been recruited.

## Abbreviations

ACG: American College of Gastroenterology; ALT: Alanine aminotransferase; AST: Aspartate aminotransferase; BUN: Blood urea nitrogen; FC: Functional constipation; FGIDs: Functional gastrointestinal disorders; HCG: Human chorionic gonadotropin; ITT: Intention-to-treat analysis; PAC-QOL: Patient assessment of constipation quality of life; RCT: Randomized controlled trial; SAS: Self-rating anxiety scale; Scr: Serum creatinine; SDS: Self-rating depression scale; SF-36: Short form health survey; TCM: Traditional Chinese medicine; TPP: Treated-per-protocol analysis.

## Competing interests

The authors declare that they have no competing interests.

## Authors’ contributions

CHZ, GYH, XHX, YW, MMZ, WW, XHJ, and BZ all contributed to the development of the study protocol. CHZ drafted the manuscript, and all authors contributed to the final draft of the manuscript. All authors approved the final version.
